# A new earthworm species within a controversial genus: *Eiseniona gerardoi* sp. n. (Annelida, Lumbricidae) - description based on morphological and molecular data

**DOI:** 10.3897/zookeys.399.7273

**Published:** 2014-04-09

**Authors:** Darío J. Díaz Cosín, Marta Novo, Rosa Fernández, Daniel Fernández Marchán, Mónica Gutiérrez

**Affiliations:** 1 Departamento de Zoología y Antropología Física, Facultad de Biología, Universidad Complutense de Madrid, C/ José Antonio Nováis 2, 28040, Madrid, Spain; 2 Cardiff School of Biosciences, Cardiff University, BIOSI 1, Museum Avenue, Cardiff CF10, 3TL, UK; 3 Museum of Comparative Zoology, Department of Organismic and Evolutionary Biology, Harvard University, 26 Oxford Street, Cambridge, MA 02138, USA

**Keywords:** Earthworms, lumbricids, *Eiseniona*, species description

## Abstract

The morphological and anatomical simplicity of soil dwelling animals, such as earthworms, has limited the establishment of a robust taxonomy making it sometimes subjective to authors’ criteria. Within this context, integrative approaches including molecular information are becoming more popular to solve the phylogenetic positioning of conflictive taxa. Here we present the description of a new lumbricid species from the region of Extremadura (Spain), *Eiseniona gerardoi* **sp. n.** The assignment to this genus is based on both a morphological and a phylogenetic study. The validity of the genus *Eiseniona*, one of the most controversial within Lumbricidae, is discussed. A synopsis of the differences between the type species and the west-European members of the genus is provided.

## Introduction

Earthworm fauna is still poorly known within vast areas of the Iberian Peninsula. The available data indicate the common presence of cosmopolitan species such as *Aporrectodea trapezoides* (Dugès, 1828) or *Aporrectodea rosea* (Savigny, 1826). In contrast, other species show more restricted distributions but are locally abundant (Díaz Cosín et al. 1992, Rodríguez et al. 1997). The region of Extremadura is one of the best documented, thanks to the work by Sánchez et al. (1998, 1999). These authors found that *Aporrectodea trapezoides* and *Aporrectodea rosea* are the dominant species, while other species can be locally important in river sides and flooded areas, such as the species of the complex *Allolobophora molleri* Rosa, 1889 sensu Barros et al. (1992) that was placed in *Eophila* by Blakemore (2008).

An intensive earthworm sampling campaign was accomplished between 2009 and 2012 in the surroundings of Plasencia (North of Cáceres, Extremadura, Spain) within the European Project “BioBio, Biodiversity Indicators for European Farming Systems, Indicators for Biodiversity in Organic and Low Input Farming Systems”. The Spanish team within this project studied the potential use of soil fauna as bioindicators in *dehesas* (i.e., Mediterranean grazed open woodlands of *Quercus ilex* Linné and olive groves under different types of land management. Among the several thousands of earthworm specimens collected during this sampling campaign, nineteen individuals sampled close to El Bronco (Cáceres, Spain) are of special taxonomical interest as they represent a new species as described in the present study.

The taxonomical assignment to a genus level in earthworm lumbricid taxonomy is confusing and varies regarding the criteria used by the different authors. In addition, it lacks robustness because it is not necessarily based on phylogenetic relationships. The number of genera proposed for the family Lumbricidae varies from five when reviewed by Michaelsen (1900) (*Eiseniella*, *Eisenia*, *Helodrilus* – with four subgenera – *Octolasium* and *Lumbricus*) to 44 proposed by Blakemore (2008) or 45 considered by Qiu and Bouché (1998a), including 29 subgenera. Some of these genera are well-defined and characterized by consistent and stable characters. A good example is the genus *Lumbricus*, with a tanylobic prostomium, paired chaetae and reddish body colour. Unfortunately, this is not the case in the great majority of the other genera, as often overlapping or slightly variable characters are used to define them. Therefore, the proper assignment to the level of genus is challenging and sometimes even subjective, but should nevertheless comply with ICZN requirements to be consistent with its type-species.

Soil dwelling animals are subject to a series of limitations in their corporal design. This is reflected in earthworms that present a very simple body externally without many differential morphological characters. The position of clitellum and tubercula pubertatis, type of prostomium, pigmentation, chaetal arrangement, number and position of spermathecae, seminal vesicles, Morren’s glands, nephridia or typhosole are some of the most widely-used morphological characters in earthworm systematics. Nevertheless, these characters may probably have evolved as adaptations to a particular soil environment or independently in several phylogenetic lineages, therefore hindering establishment of a robust taxonomical system based on morphology. The solution to this taxonomical chaos would be the phylogenetic resolution of earthworms based on molecular and morphological studies. This would allow the generation of stable and robust phylogenies in which systematic classifications are properly defined. Unlike earthworms from the family Hormogastridae (e.g., Novo et al. 2011), only some attempts have been done to study the phylogenetic relationships of lumbricids (e.g., Briones et al. 2009). Nonetheless, these studies are limited because of incomplete sampling or limited number of genes sequenced. Fortunately, a multigene phylogeny of lumbricid earthworms represented by a high number of species and genera is about to be published (Pérez Losada et al., *pers. comm.*) and hopefully it will help to convert lumbricid systematics into a more stable system.

In the context of this controversial classification of genera in lumbricid earthworms, one of the most conflictive ones is *Eiseniona* (Omodeo, 1956). This genus was established by Omodeo (1956) and was accepted by some authors (Álvarez 1970; Qiu and Bouché 1998a, d; Omodeo and Rota 2004; Rota 2013) but rejected by others (Bouché 1972; Zicsi 1982; Easton 1983; Mrsic 1991 and Blakemore 2008). Most of the species included in *Eiseniona* are distributed in Italy, Greece and other countries of Central or Eastern Europe. Some examples of species described in Western Europe are *Eiseniona paradoxa* (Cognetti, 1904) and *Eiseniona gavarnica* (Cognetti, 1904) in France [both retained in genus *Orodrilus* Bouche, 1972 by Blakemore (2008)] and in the Iberian Peninsula *Eiseniona oliveirae* (Rosa, 1894), *Eiseniona carpetana* (Álvarez, 1970) and *Eiseniona albolineata* Díaz Cosín et al., 1989 [the former retained in *Allolobophora* Eisen, 1874 and the latter two retained in genus *Iberoscolex* Qiu & Bouche, 1998 by Blakemore (2008)].

Despite the extended use of morphological and anatomical characters in earthworm taxonomy, during the last years the concept of integrative taxonomy as a tool to describe and delimit species has become more popular. This concept, consisting of a multidisciplinary approach including the morphological, molecular, ecological and geographical available data, has been applied to earthworms (e.g., Novo et al. 2012 for hormogastrids, Blakemore and Kupriyanova 2010, Blakemore 2010, Blakemore et al. 2010, Blakemore and Grygier 2011 and Blakemore 2012a for lumbricids) The implementation of molecular techniques has allowed presumption of a high cryptic diversity in earthworms completely unknown when based on traditional systematic methods (e.g. King et al. 2008, Novo et al. 2009, 2010, Dupont et al. 2011, Fernández et al. 2011), but see critique in Blakemore et al. (2010). In addition, molecular barcoding has become a widely used technique for taxonomical evaluation, allowing interesting discoveries such as the proposed separation of *Lumbricus terrestris* and *Lumbricus herculeus* (James et al., 2010), but see correction by Blakemore (2013).

In this context, this manuscript aims to describe a new lumbricid species (*Eiseniona gerardoi* sp. n.) based on morphological, molecular and ecological data.

## Material and methods

### Earthworm specimens and sampling points

**Figure 1.**
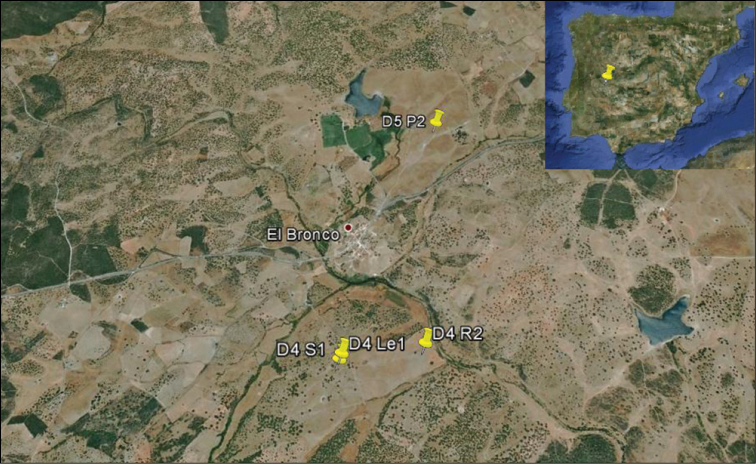
Map showing the position of sampling points.

Nineteen individuals were collected at four different but geographically-close sampling points nearby El Bronco (Cáceres, Extremadura, Spain). Soil was a sandy-loam on underlying slate (Figure 1); collectors G. Moreno, E. Juárez.

D4 Le1: **2 ex.** (1 adult, 1 subadult) (40°12'42.76"N, 6°19'0.68"W). Altitude 430 m. Grazed *dehesa* with *Quercus ilex*. Mean precipitation 876 mm. Present plant species: *Eleocharis palustris*, *Pulicaria paludosa*. Other earthworm species: *Allolobophora molleri* 1 ex. (0.75 g).

D4 R2: **2 ex.** (2 adults) (40°12'45.22"N, 6°18'39.22"W). Altitude 414 m. Grazed *dehesa* with *Quercus ilex*. Mean precipitation 876 mm. Present plant species: *Anthoxanthum aristatum*, *Isoetes hystris*. Other earthworm species: *Allolobophora molleri* 8 ex. (6.72 g), *Aporrectodea trapezoides* 16 ex. (4.96 g).

D4 S1: **2 ex.** (2 subadults) (40°12'41.51"N, 6°19'1.20"W). Altitude 430 m. Grazed *dehesa* with *Quercus ilex*. Mean precipitation 879 mm. Present plant species: *Festuca ampla*, *Trifolium dubium*. Other earthworm species: *Allolobophora molleri* 2 ex. (2.02 g), *Aporrectodea trapezoides* 3 ex. (2.01 g).

D5 P2: **13 ex.** (5 adults, 8 subadults) (40°13'38.80"N, 6°18'36.04"W). Altitude 428 m. Grazed *dehesa* with *Quercus ilex*. Mean precipitation 923 mm. Present plant species: *Juncus bufonius*, *Conyza* sp. Other earthworm species: *Allolobophora molleri* 6 ex. (5,32 g), *Aporrectodea rosea* 4 ex (1.05 g), *Aporrectodea trapezoides* 32 ex (18,91 g).

### Molecular sequencing and analyses

The following molecular regions were amplified by the methods described in Novo et al. (2011): mitochondrial subunit I of cytochrome c oxidase (COI), 16S rRNA and tRNA Leu, Ala, and Ser (16S-tRNAs), two nuclear ribosomal genes (complete 18S and a portion of 28S) and two nuclear protein-encoding genes (histones H3 and H4).

In order to have an evaluation of the selection of species to include in the molecular analyses, M. Pérez-Losada and J. Domínguez (Universidad de Vigo) kindly compared the sequences of 16S and 28S rRNA from the specimens included in this study with an unpublished database that includes most lumbricid genera. This comparison provided the first evidence indicating that the new species was closely related to *Eiseniona albolineata* and *Eiseniona oliveirae*. As a second method, we collected some individuals belonging to *Eiseniona albolineata* and sequenced the mitochondrial gene COI. In addition, we retrieved from GenBank all available COI sequences from as many different lumbricid species as possible to date (Table 2), although many of these have their identities unconfirmed. We excluded from the analyses the sequenced genes in the public databases for which information is scarce and biased. Bayesian phylogenetic inference was then explored with the COI sequences as described in Fernández et al. (2012).

Uncorrected pairwise differences were calculated between these species with Arlequin 3.5 (Excoffier et al. 2005).

## Data resources

The data underpinning the analysis reported in this paper are deposited in the Dryad Data Repository at http://dx.doi.org/10.5061/dryad.5k76c

## Results

The specimen with voucher number UCMLT 60000 is the designated holotype. The paratypes bear the numbers UCMLT 60001 to 60018.

### Morphological description

The specimens were sketched using an Olympus binocular microscope with digital camera, dissected, and described.

### Taxonomic results
Phylum Annelida Lamarck, 1802
Subphylum Clitellata Michaelsen, 1919
Class Oligochaeta Grube, 1850
Order Haplotaxida Michaelsen, 1900
Family Lumbricidae Rafinesque-Schmaltz, 1815

Genus *Eiseniona* Omodeo, 1956

**Type-species.** *Allolobophora handlirschi* Rosa, 1897 by original designation.

#### Eiseniona gerardoi

Díaz Cosín sp. n.

http://zoobank.org/E14BF86D-EFF1-47E7-BE5B-6F59ACCDCD4B

http://species-id.net/wiki/Eiseniona_gerardoi

##### Material examined.

*Holotype*. Adult (Catalog # UCMLT 60000), 40°13'38.80"N, 6°18'36.04"W (“spanish dehesa” mediterranean grazed open woodlands of *Quercus ilex*), near El Bronco (Cáceres, Spain), leg. G. Moreno, E. Juárez, April 2010.

*Paratypes*. 18 specimens (Catalog # UCMLT 60001 to 60018), leg G. Moreno, E. Juárez, April 2010.

##### Morphological description.

**Figure 2.**
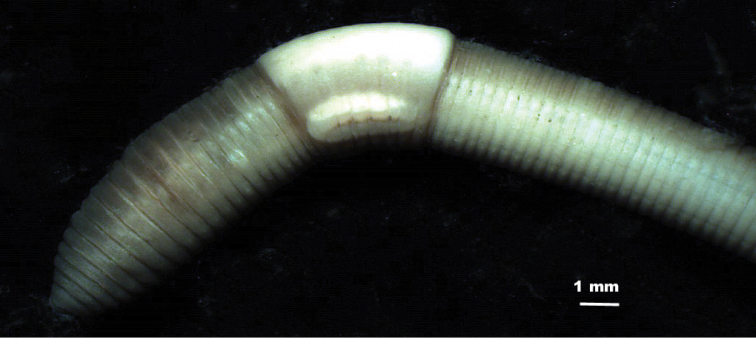
External view of the anterior part of the body of *Eiseniona gerardoi*.

**Figure 3.**
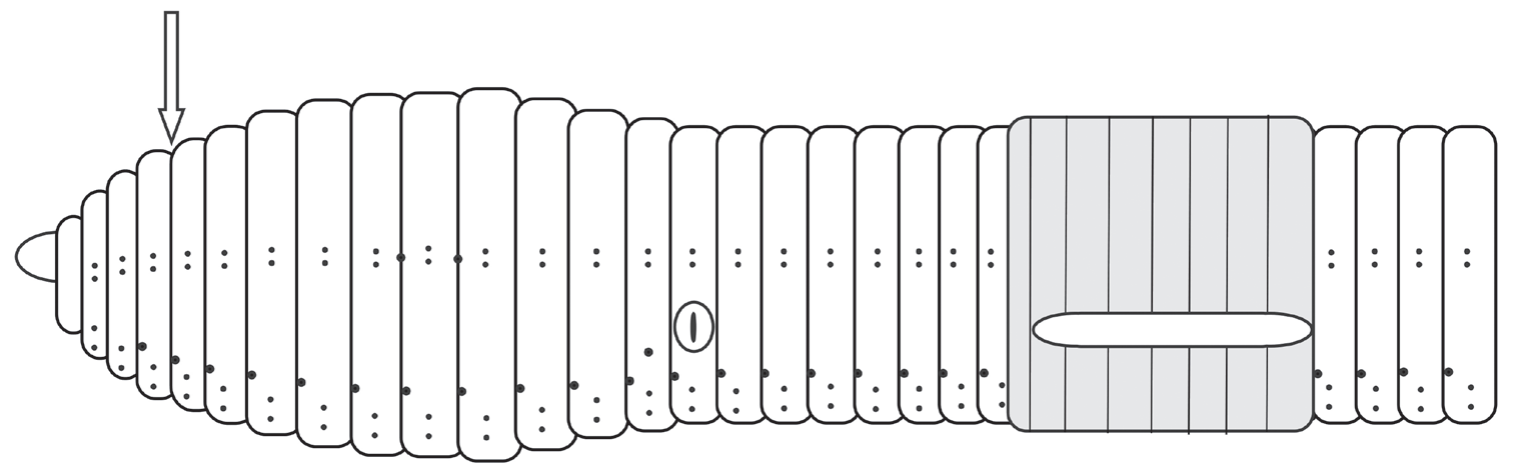
Schematic view of the external morphology of *Eiseniona gerardoi*.

*External morphology* (Figures 2, 3). Length of mature specimens: 21–40 mm, x: 28 mm, SD: 5.6 mm, holotype: 31 mm. Diameter: clitellar x: 2.5 mm, SD: 0.4 mm, holotype: 2.5 mm, postclitellar x: 1.8 mm, SD: 0.2 mm, holotype: 1.7 mm. Body cylindrical in the anterior part, wider at clitellum and trapezoidal or rectangular in postclitellar region, with chaetae in the corners. Number of segments: 89 to 124, x: 109.5, SD: 10.7, holotype: 117. Weight (fixed specimens): 38 to 64 mg, x: 52 mg, SD: 13 mg, holotype 62 mg.

Colour: When alive, the anterior part is red-brownish showing noticeable antero-posterior and dorso-ventral gradients. Cream-coloured or whitish clitellum. After a long period within alcohol the red pigment is gradually lost and transformed into brown of different intensities (Figure 2).

Prostomium epilobic ±1/3. No longitudinal lines are noticeable in segments 1 and 2. First dorsal pore in (3/4) 4/5. Nephridial pores inconspicuous in a row slightly above *b.* Spermathecal pores at intersegments 9/10 and 10/11, at the level of chaetae *cd* (Figure 3).

**Figure 4.**
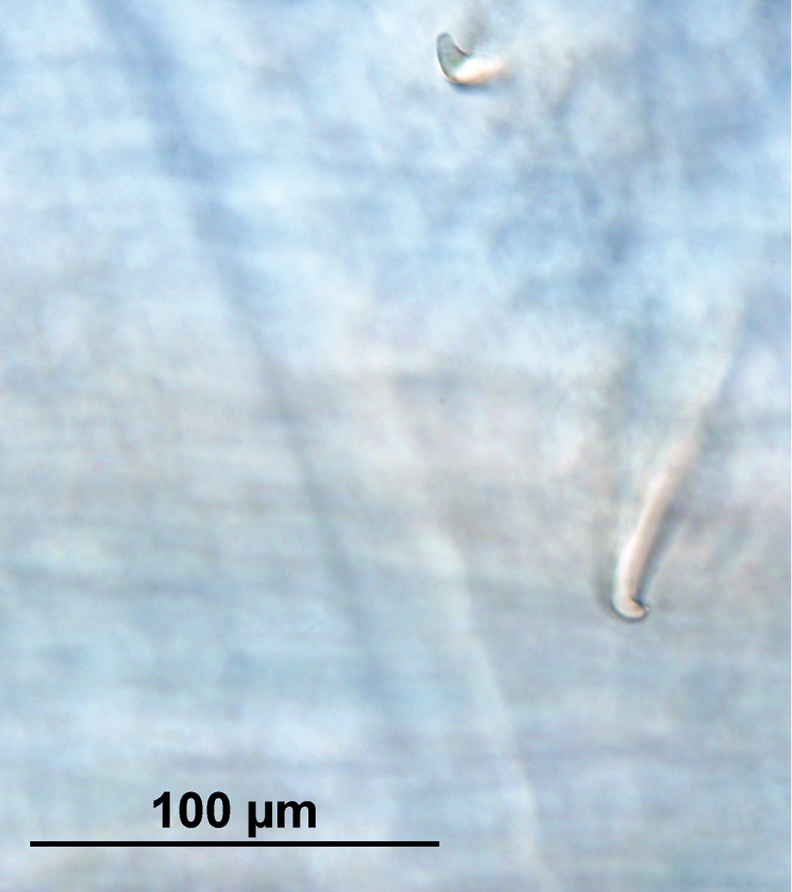
Chaetae *ab* from segment 40–41 (DIC Nomarski).

Male pores as vertical grooves in the segment 15 between chaetae *b* and *c* showing small porophores with whitish areolae shape. Female pores in 14 slightly above *b.* Chaetae paired, interchaetal ratio at segment 40, *aa*: 16, *ab*: 1.4, *bc*: 7, *cd*: 1, *dd*: 24. Chaetae are simple with a wider base and a sharp and bent distal end. (Figure 4).

Clitellum white or cream-coloured, saddle-shaped extending over 22,23–29,30, in the holotype 1/n 22, 23–29. When well developed it invades the ventral area and the intersegmental lines are hard to distinguish. Tubercula pubertatis extended as a belt in 23-(27)28,29, in the holotype in 23–29. Occasionally they appear folded or wrinkled. No noticeable papillae are present in any of the specimens.

*Internal anatomy*. Slightly thickened anterior septa. Last pair of oesophageal hearts in 11. Morren’s glands with small diverticula in 10 and little lamellae in 11 and 12. Crop in 15,16, gizzard in (17)18,19. First section of the intestine is not dilated. Simple typhlosole pleated, which begins in 20, 21 and ends near the anus leaving only 10–15 atyphlosolate segments.

Fraying testes and iridescent and very large seminal funnels in 10 and 11. Three pairs of seminal vesicles in 9, 11 and 12. The last pair is very large pushing back the septum 12/13. Large ovaries and female funnels in 13, ovarian receptacles (ovisacs) in 14. Two pairs of very large and iridescent spermathecae in segments 10 and 11.

**Figure 5.**
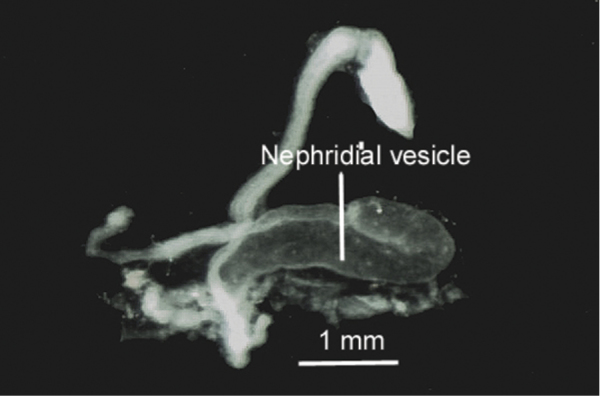
Posterior nephridium isolated by dissection, showing the nephridial curved bladder.

In the posterior region of the body the nephridia are much enlarged, the nephridial bladders are curved and J-shaped with curved section 1/3 of total length. (Figure 5).

**Figure 6.**
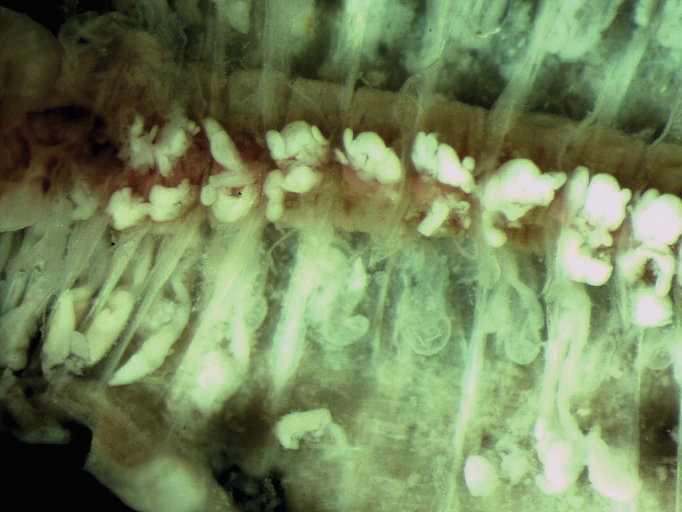
White tissue associated with the dorsal vessel.

An important characteristic is the presence of dense white glands on top of the dorsal vessel initially around segment 20 and externally visible as a whitish line extending to the end of the body. (Figure 6).

##### Distribution.

Known only from its type locality.

##### Etymology.

The species is dedicated to Prof. Gerardo Moreno from Centro Universitario de Plasencia, Universidad de Extremadura, Spain. He is the PI for the Bio-Bio program in Spain and collected the specimens described in this paper.

##### Molecular characters.

Sequences of the used genes have been deposited in GenBank (see Table 1). According to Drs. Pérez Losada and Domínguez (*pers. comm*.), the 16S and 28S sequences of *Eiseniona gerardoi* clustered with the two species classified as *Eiseniona*, *Eiseniona albolineata* and *Eiseniona oliveirae*.

**Table 1.** Paragenetypes (sensu Chakrabarty 2010) of *Eiseniona gerardoi* sp. n. paratypes, with GenBank accession numbers. As a consequence of the UCM scientific collections restructuring, the earthworms are now deposited within UCMLT (Universidad Complutense de Madrid Lombrices de Tierra).

Voucher	COI	16S-tRNAs	18S rRNA	28S rRNA	H3	H4
UCMLT 60001	KF737142	KF737134	KF737140	KF737148	KF737150	HG780373
UCMLT 60002	KF737143	KF737135	KF737141	KF737149	KF737151	HG780374
UCMLT 60007	KF737144	KF737136			KF737152	HG780375
UCMLT 60013	KF737145	KF737137				
UCMLT 60015	KF737146	KF737138				
UCMLT 60017	KF737147	KF737139				

**Table 2.** Taxa and specimens included in the molecular analysis. GenBank accession numbers are indicated. Species names were literally taken from GenBank. The correct name [after Blakemore (2008)], of the species marked with asterisk is, *Bimastos parvus*, *Allolobophoridella eiseni* and *Iberoscolex albolineatus*.

Species	COI GeneBank accession number
*Allolobophora chlorotica*	GU013806
*Aporrectodea longa*	JN850544
*Aporrectodea rosea*	FJ214232
*Aporrectodea trapezoides*	JF313567
*Aporrectodea tuberculata*	JN869877
*Bimastus parvus	EF077605
*Dendrobaena attemsi*	FJ214224
*Dendrobaena octaedra*	GU013836
*Dendrobaena veneta*	FJ214233
*Dendrodrilus rubidus*	GU013839
*Eisenia andrei*	DQ914619
*Eisenia eiseni	AY874488
*Eisenia fetida*	EF077596
*Eiseniona albolineata	KF746384
*Helodrilus oculatus*	FJ374775
*Hormogaster elisae*	EF653905
*Lumbricus festivus*	FJ937290
*Lumbricus rubellus*	GU206189
*Lumbricus terrestris*	JN869936
*Octodrilus juvyi*	HE611693
*Octolasion cyaneum*	JQ909144
*Octolasion lacteum*	DQ092909

**Figure 7.**
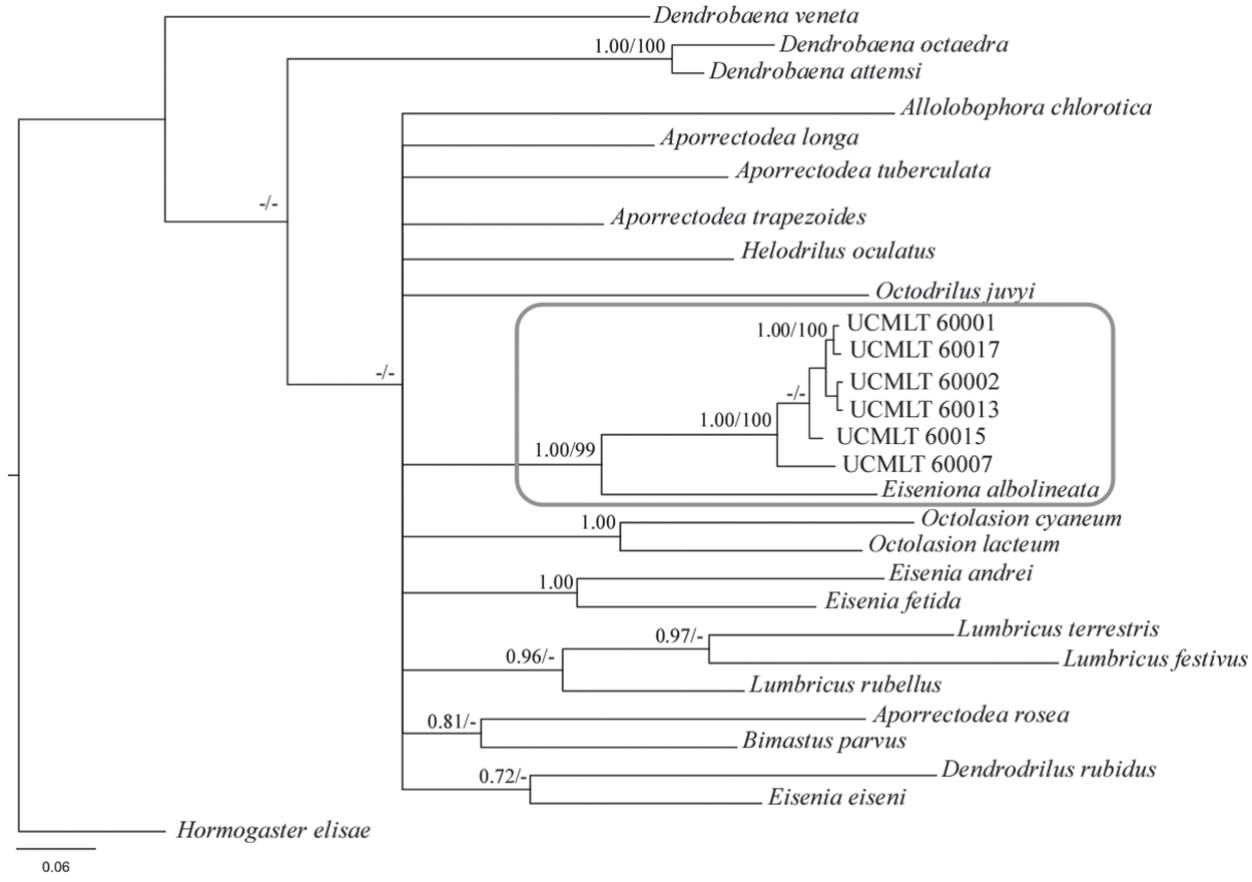
Bayesian inference tree based on COI sequences of *Eiseniona gerardoi* and other lumbricids represented in GeneBank. *Eiseniona gerardoi* (see UCMLT codes in Table 1) clusters with *Eiseniona albolineata*.

The phylogenetic tree presented here, based on the COI gene and including some of the available species in GenBank (Figure 7), shows that *Eiseniona gerardoi* specimens form a highly supported group (1.00 posterior probability, 0.99 bootstrap) with *Eiseniona albolineata*. The two species share the presence of whitish glands on top of the dorsal vessel. COI genetic divergence (uncorrected p-distances) between *Eiseniona albolineata* and *Eiseniona gerardoi* is 14.09%, and the intraspecific variability of the latter is 2.81% showing a very close relationship.

##### Habitat and ecological characters.

All the soils from sampling sites have been developed on slates and are sandy-loams. Precipitation corresponds to the typical values of intermediate semi-humid Spain. The associated species *Allolobophora molleri* is always present and this species is bound to terrains that are flooded during several months per year. Additionally, the presence of plants typical from wetlands, such as *Eleocharis palustris*, *Pulicaria paludosa* or *Juncus bufonius* indicates that in these sites there is enough humidity during most of the year, which supports hygrophile communities. Nevertheless they could be desiccated in the summer, which would force the earthworms to undergo aestivation in order to survive to these dry periods, resuming activity when humidity is restored. All these details are compatible with the diagnosis of the genus by Omodeo (1956) as he highlighted the semiaquatic characteristics of *Eiseniona*.

## Discussion

*Eiseniona* genus was created by Omodeo (1956) for the inclusion of five earthworm species presenting small to medium sizes, number of segments inferior to 170, closely paired chaetae, Morren’s glands with lateral bags in segment 10 and red or light pink subepidermic pigment (absent in some instances). Their clitella start between segments 23 and 27 and tubercula pubertatis appear as continuous bands. Male pores lack the glandular atrium (but show a small atrium in some instances) and papillae are present in segments near the spermathecae and male pores. They show three or four pairs of seminal vesicles, the last reaching to segments 13 or 14. Their habitat is semiaquatic. Omodeo and Rota (2004) subsequently added or specified other characters such as: “body central and posterior parts with trapezoidal cross section, with the pairs of chaetae at the four corners, nephridial bladders as an inverted J with the ental limb oriented backward, typhlosole cylindrical “en accordéon” spermathecae large, intracoelomic with openings in 9/10 and 10/11, three pairs of seminal vesicles in IX, X, XI the latter being very large, protruding in XIII”.

The species originally included in this genus were *Eiseniona handlirschi* (Rosa, 1897) [the designated type, now placed in *Aporrectodea* according to Blakemore (2008) and Csuzdi (2012)], *Eiseniona oliveirae* (Rosa, 1893), *Eiseniona intermedia* (Michaelsen, 1901), *Eiseniona paradoxa* (Cognetti, 1904) and *Eiseniona sineporis* (Omodeo, 1952). Two new species from Spain were included afterwards, *Eiseniona carpetana* Álvarez, 1970 and *Eiseniona albolineata* Díaz Cosín et al. 1989, Qiu and Bouché (1998a, d) accept the genus *Eiseniona* in which they include 17 species or subspecies, most of them distributed in the Balkans. However, they place *albolineata* and *carpetana* within the genus *Iberoscolex*; *gavarnica* and *paradoxa* within *Orodrilus* and *oliveirae* within *Koinodrilus* Qiu and Bouché (1998b, c). The diagnosis of *Eiseniona* by these authors is slightly different from the one by Omodeo and Rota (2004), mainly regarding details such as pigment absence, pinnate typhlosole or the presence of four pairs of seminal vesicles in 9, 10, 11 and 12.

Blakemore (2008) did not accept the genus *Eiseniona* and considered it as a synonym of *Aporrectodea*. This author highlighted that it was neither accepted by Bouché (1972), who included hemiandric forms such as *paradoxa* and *gavarnica* within the genus *Orodrilus* and the remainder within *Allolobophora*. Neither was it accepted by Zicsi (1981, 1982b) nor Mrsic (1991), who note that “the diagnosis of this genus is deficient (the species are hard to distinguish from those of the genus *Aporrectodea*), so I ignore it.” It is evident that the validity of *Eiseniona* is controversial and in this sense Blakemore (2008) stresses that ”These issues need to be thoroughly resolved, with reference to types, before we can consider restoration of either *Eiseniona* or *Koinodrilus*”.

Phylogenies recovered by molecular methods can aid to solve this problem by providing key information to support systematics and therefore approaching a natural system (Novo et al. 2011). On this topic Blakemore (2012a) states the need of basing the molecular analyses on the types of the genera. A molecular comparison with the type species *Eiseniona handlirschi* could not be carried out in this study due to lack of material. However, in the phylogenetic trees we present here, *Eiseniona gerardoi* clustered together with *Eiseniona albolineata* and it is clearly differentiated from the other genera. The assignment of this new species to the genus *Eiseniona* is further supported by the fact that analyses with 16S and 28S regions place it near *Eiseniona albolineata* and *Eiseniona oliveirae* within an unpublished phylogeny of lumbricids that includes most of their genera (Pérez Losada and Domínguez *pers. comm.*). Hence, the new species can be located within an explicit phylogenetic context, near *albolineata* and *oliveirae* regardless of their generic assignment.

Some of the features of our specimens, such as the lack of papillae or the presence of porophores in segment 15, are different from the ones described for most *Eiseniona*. However male porophores of *Eiseniona gerardoi* are relatively small and Omodeo’s (1956) indicates in its diagnosis that in some instances small porophores might be present in the genus. Apart from that, most of the traits of *Eiseniona gerardoi* are compatible with those originally diagnosed as the generic features of *Eiseniona*. Moreover *Eiseniona gerardoi* shares with *Eiseniona albolineata* the white tissue developed on the dorsal vessel.

Considering all this data, we opt to include this new species, at least provisionally, within *Eiseniona* because it is the less troublesome position within the current genera system for Lumbricidae. This is suggested not only by morphological and ecological considerations but also by the molecular data placing it near *Eiseniona albolineata* and *Eiseniona oliveirae*.

The phylogeny of species historically included within *Iberoscolex*, *Koinodrilus* and *Eiseniona* will need to be thoroughly revised in the future, in order to clarify whether they represent good genera and to find a robust grouping of the species within genera, which does not seem possible exclusively with morphological tools. It is also noteworthy that within *Eiseniona* there is a group of species from Southern France and Iberian Peninsula and another one from Italy, Greece and Central and Eastern Europe. Future studies will unravel whether these two groups constitute independent phylogenetic units susceptible to be taxonomically divided.

A considerable effort is still necessary to establish a robust genera system based on phylogeny within lumbricids. This system should integrate the study of mitochondrial and nuclear markers with morphological characters and include representatives from all the proposed genera and type species. Until the moment when such big picture is available controversy on lumbricids’ genera system will continue and different authors will apply subjective criteria.

### Differences with other species of the genus

The most similar species to *Eiseniona gerardoi* regarding clitellum position and tubercula pubertatis is *Eiseniona intermedia*, but the last has a much greater size, its tubercula pubertatis start in a more posterior segment and presents four pairs of seminal vesicles. In addition, it was only found in Bashkiria (Bashkortostan, Russia) (data from Omodeo 1956). The differences of *Eiseniona gerardoi* with the remaining species included within *Eiseniona* by Omodeo (1956) and Qiu and Bouché (1998a, d) are clear in terms of the beginning of clitellum in segments 22,23 and the tubercula pubertatis in segment 23. A comparison of some characters of the species living in the western part of the geographic range of *Eiseniona* is shown in Table 3, excluding the hemiandric *Eiseniona paradoxa* and *Eiseniona gavarnica*.

**Table 3.** Comparison of species living in the western part of the geographic range of *Eiseniona*. The type species *Eiseniona handlirschi* is included and the hemiandric *Eiseniona paradoxa* and *Eiseniona gavarnica* are excluded.

	*Eiseniona albolineata*	*Eiseniona carpetana*	*Eiseniona oliveirae*	*Eiseniona gerardoi*	*Eiseniona handlirschi*
Length (mm)	78–122 matures	52–74	85–110 * 30–86** 45***	21–40	50–60* 50–170** 50–95***
Segments	138–172	129–150	167 * (77) 100–131** 125***	89–124	120–130* 115–163** 78–119***
Colour	Grey, posterior white line	Rose violet	Light flesh tone* Brown or violet “in vivo”, greyish when fixed** Brown, red***	Red-brownish “in vivo”, posterior white line	Colourless* Colourless** Pale reddish***
Chetae	Separate 2.5 - 1.2 - 2.2 - 1 - 5	Separate	Closely paired* 6.7 – 1.3 – 6.2 – 1 – 11.8** Closely paired 9 – 1.5 – 7.5 – 1 - 18***	Paired 16 - 1.4 - 7 - 1 - 24	Closely paired 8 – 1.15 – 6 – 1 – 20***
First dorsal pore	(4/5) 5/6	4/5	4/5* (4/5) 5/6** 5/6***	(3/4) 4/5	From 4/5, usually 19/20** 17/18 to 23/24***
Spermathecae	10,11, pores 9/10, 10/11 near *d*	10, 11, pores 9/10, 10/11 *c*	10, 11, pores 9/10 10/11 near *c*	10, 11, large, iridescent, pores 9/10, 10/11 *cd*	9, 10, pores in 9/10 10/11
Clitellum	(24)25 – 30(31)	Annular in (1/2 24)25 -1/2 31(31)	24–30* (23)24–29(30)** 24–29***	22,23–29,30	26–33* (25,26)27–32(33)** 25,26(27)-(32)33***
T. pubertatis	1/n 26 – 28(1/2 29)	25–30	24–30* 24–29,30** 1/2 25–28***	23-(27)28,29	29–32* (1/2 27,28)29–30 (31,32)** 1/n 28–31,32***
Gld. Morren	10–12, diverticula in 10	11- 12, no diverticula	10–13 diverticula in 10** 11–14, no diverticula***	10,11,12 small diverticula in 10	Diverticula in 10 10–13***
Nephridial vesicle	S - shaped	?	Curved, reclined***	J - shaped	Inverted J***
Typhlosole	Bifid initially, later simple	?	Simple	Simple, pleated	Circular, transversally pleated***
Seminal vesicles	9,10,11,12	9,10,11,12	9,11,12* 9, 10,11,12** 9,11,12***	9,11,12	9,11,12* 9,(10),11,12** 9,11,12***
Others	White tissue on top of the dorsal vessel.		*Rosa (1894) ** Díaz Cosín et al. (1985) *** Qiu and Bouché (1998b)	White tissue on top of the dorsal vessel	*Rosa (1897) ** Bouché (1972) *** Omodeo and Rota (2004)

Genetic divergence between *Eiseniona gerardoi* and *Eiseniona albolineata* (COI, uncorrected distances) is 14.09%, which is within the interval of uncertainty proposed by Chang and James (2011), but still near the 15% that these authors consider as indicative for different species in earthworms. Nevertheless there are enough morphological characters that permit the separation of the two species.

## Supplementary Material

XML Treatment for Eiseniona gerardoi
